# Interventions for adults with deafblindness - an integrative review

**DOI:** 10.1186/s12913-022-08958-4

**Published:** 2022-12-30

**Authors:** Camilla Warnicke, Moa Wahlqvist, Agneta Anderzén-Carlsson, Ann-Sofie Sundqvist

**Affiliations:** 1 University Health Care Research Centre, Faculty of Medicine and Health, Örebro University, Örebro, Sweden; 2 Audiological Research Center, Faculty of Medicine and Health, Örebro University, Örebro, Sweden; 3 The Swedish National Resource Center for Deafblindness, Lund, Sweden

**Keywords:** Communication, Deafblindness, Dual sensory loss, Information, Intervention, Orientation, Rehabilitation

## Abstract

**Purpose:**

To compile the current research on interventions for rehabilitation aimed at adults (aged 18-65 years) with deafblindness.

**Materials and methods:**

A comprehensive search was conducted in eight databases. An additional manual search was also carried out. A total of 7049 unique references were initially identified, and after screening, 28 original scientific articles were included. The results from these articles were categorized based on limiting consequences of deafblindness: communication, orientation and to move around freely and safely and access to information, as well as to psychological adaptation to deafblindness.

**Results:**

Fourteen of the included articles had their main focus on access to communication, ten on orientation and the ability to move around feely and safely, three on the opportunity to gain access to information, and one related to psychological adaptation to deafblindness. Most articles focused on technical devices, of which one-third were single case studies.

**Conclusion:**

There is a limited number of evaluated interventions for people with deafblindness. Most of the existing studies involved one to five participants with deafblindness, and only few studies involved a larger number of participants. More research with a larger number of participants are needed, which could be facilitated by international cooperation between practitioners and researchers.

**Supplementary Information:**

The online version contains supplementary material available at 10.1186/s12913-022-08958-4.

## Introduction

People with deafblindness (DB) are identified as an especially vulnerable group [1, 2]. DB is defined as a “combination of vision and hearing impairment of such severity that it is hard for the impaired senses to compensate for each other” [3]. In 2018, approximately 0.2-2 % of the world’s population were living with the limitations of combined hearing and vision impairment (i.e., DB) [1]. However, the range of prevalence among populations, studies or countries, as well as across age groups, and types of DB varies [4]. There are many causes of DB, which may be present from birth (i.e., congenital DB; CDB) or acquired later in life [5, 6]. When having CDB, the vision and hearing impairment is established before the development of language, while acquired deafblindness appear after the development of language (spoken or sign language). Although the World Federation of the Deafblind, in line with the Nordic Definition [3] define deafblindness as a distinct disability, they also state that the people with DB is a heterogeneous group, were some are completely deaf and blind, and others have a little sight and/or hearing they can use [1]. This notion is underscored by Deafblind Ontario Services, who emphasize that each affected person has their unique experience of the decline in vision and hearing, which individually affects their access to information, and their ability to participate and being included in the community [7]. Regardless of the cause and degree of sensory loss, living with DB affects many aspects of everyday life. The effects of DB include communication challenges, restriction of daily living activities, risk of isolation, lack of independence, and difficulty in orientation and the ability to move around freely and safely [2, 8, 9]. Additionally, poor health outcomes may result in deficits in psychological and physical health [10–12] and exposure to stress [13]. The implications of this disability require communities to facilitate the provision of services and adjustments to the environment and/or technology to meet the needs of people with DB to afford them full inclusion in the activities of everyday life [1].

There are challenges with regard to receiving appropriate formal support, i.e., support from health-care or rehabilitation departments, for people with DB [5]. There may also be a lack of professional knowledge on the part of others regarding the disability, with individuals reporting that they often have to educate officials or other personnel themselves, which leads to frustration and exhaustion [14]. There is a need for a focus on interventions that enhance participation in daily activities for people with DB [15]. Interventions can be seen as a process with the purpose to enable people with DB to establish and maintain control over the environment at a level appropriate to their functioning, and the interventions shall be defined by the needs of the people with DB themselves [16]. Several articles have addressed the need for tailored interventions for individuals with DB as well as for their family members [17–19].

The World Health Organization defines rehabilitation as “a set of interventions designed to optimize functioning and reduce disability in individuals with health conditions in interaction with their environment” [20]. The Swedish National Resource Center for Deafblindness has addressed the need for knowledge on existing rehabilitation interventions conducted in research and practice for adults with DB. To the best of our knowledge, there is no international compilation of interventions focusing on DB that are evaluated. Such a review could form the basis for evidence based guidelines or serve as an inspiration for clinicians working with this group of individuals. It could also identify knowledge gaps.

The aim of this review was to compile the current research on interventions for rehabilitation aimed at adults (aged 18-65 years old) with DB.

## Materials and methods

### Design

This article is an integrated review outlined in accordance with Whittemore and Knafl [21]. The method involves the summation of previously conducted empirical and theoretical literature with the possibility of incorporating different methodologies, such as both qualitative and quantitative methods, to capture the topic [21, 22], which may contribute to a broader and deeper understanding of interventions for people with DB [23].

### Literature search strategy

A comprehensive database search to identify peer reviewed articles that focus on interventions related to the consequences of DB which have been evaluated by either people with DB themselves, their relatives or professionals, was conducted. Search terms and search strategies were developed together with a subject specialist librarian using the SPICE framework [24]; see Table 1. Two experienced librarians conducted the search in a total of eight databases: AMED, Cinahl, Embase, ERIC, PsycINFO, PubMed, Scopus, and Web of Science. The databases were searched for original scientific articles published in Danish, English, Norwegian or Swedish.

**Table 1:** Overview of the purpose and inclusion criterions

Purpose: To compile the current research on interventions for rehabilitation aiming at adults with deafblindness (DB)				
Setting	Perspective	Intervention	Comparison	Evaluation
Where?	For whom?	What?	Compared with what?	With what result?
All countries All contexts Original scientific studies published between 2000 and 2020	Adults (18-65 years) with DB, their relatives, and professionals The DB can be congenital or acquired, and it does not need to be determined on the basis of objective criteria Studies with mixed age populations where the results for adults with DB, their relatives, or professionals working with them can be distinguished from other results	Interventions related to DB specific difficulties of relevance for rehabilitation services Interventions for people with DB, their relatives and professionals Interventions in the surrounding environment	Comparisons not necessary	The result can be: perceived, observed, or measured The result can be described from one of the following perspectives: the people with DB, their relatives, or professionals

An extensive number of search terms were used and combined with the Boolean operators AND/OR when performing the searches. The search terms were related to the topic of interest covering DB, deaf-blind specific syndromes, vision, hearing, and interventions. In Table 2, examples of search terms are displayed, and the total overview of the search terms and search strategies used in each database is displayed in an additional file [see Additional file 1]. Moreover, a timespan restriction was employed to search scientific articles published from January 2000 until December 2020. The key reason for the timespan restriction was that society and technology have evolved widely and rapidly, which in our experience has had a major impact on the areas of rehabilitation for people with DB. The initial database search was carried out in December 2018 and repeated in January 2020 and 2021. The database searches that were carried out in 2018 and 2020 form the results of a Swedish report regarding rehabilitation interventions for adults with DB that was undertaken on behalf of The Swedish National Resource Centre for Deafblindness [25]. The results in that report cover a shorter timespan (2000-2019) and thus included fewer articles than the present review.

**Table 2:** Examples of search terms used in the database searches related to their topic of interest

Topic of interest	Search terms
Deafblindness	deaf-blind, deafblindness, dual sensory impairment, dual sensory loss
Syndrome	Alstrom syndrome, CHARGE syndrome, Refsum syndrome, Usher syndrome, Wolfram syndrome
Vision	retinis pigmentosa, vision impairment, vision loss, visually impaired
Hearing	deaf, hard of hearing, hearing impairment, hearing loss, impaired hearing
Intervention	cochlear implant, communication aids, haptic, interpreter, physiotherapy, rehabilitation

An additional manual search for articles was carried out. This included a manual search of the reference lists of all reviews found in the systematic search, as well as a search based on personal knowledge of the field. Additional manual searches were undertaken in the Journal of Deafblind Communication (all issues published up to 2021) and in a special issue of Frontiers in Psychology regarding Development, Wellbeing, and Lifelong Learning in Individuals with a Dual Sensory Loss [26].

### Inclusion/exclusion criteria

Articles from peer-reviewed journals that focused on evaluated interventions for adults with DB, and met the inclusion criteria are displayed in Table 1. The only criteria regarding how the intervention itself and its effectiveness was evaluated was that it had been evaluated by persons with DB, their relatives or professionals. The exclusion criteria that were applied when screening the articles are listed in Table 3.

**Table 3:** Exclusion criterions

Articles were excluded if:	
1	The described intervention and result were not clearly linked to adults with deafblindness (DB) (i.e. persons within the age span of 18-65 years)
2	The DB/combined visual and hearing impairment was solely age-related
3	The intervention was unrelated to the consequences of DB
4	The intervention was purely medical/surgical (i.e. no rehabilitation was described or following the medical/surgical intervention)
5	The intervention was evaluated by others than people with DB, their relatives or professionals working with them (i.e. persons with impaired vision or hearing only, persons without any disabilities, students)
6	They were not published in a scientific journal or had not undergone peer-review before the publication such as book chapters, conference proceedings, discussions, editorials, opinions and poster presentations
7	Published prior to January 2000
8	Published in other languages than Danish, English, Norwegian, or Swedish

### Literature search results

**Fig. 1:**
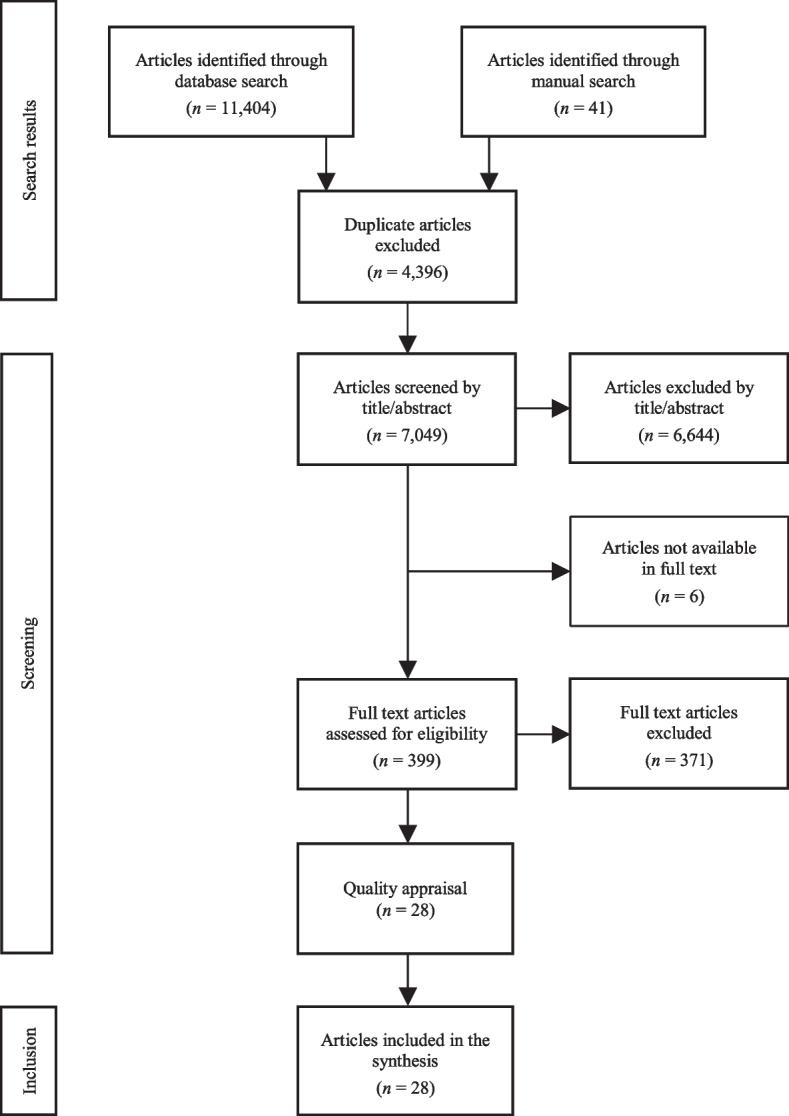
Study selection based on the PRISMA flow diagram, available from http://www.prisma-statement.org/

A total of 11,404 articles were initially identified through the combined electronic database searches, and after removing duplicates, 7008 articles remained. An additional 41 articles were found after the manual search, yielding a total of 7049 articles for assessment. The first step included a screening of the titles and abstracts for relevance wherein 6644 articles were excluded, leaving 405 articles to be retrieved in full text. Six of those could not be obtained and were therefore excluded. The full texts of the 399 remaining articles were assessed against the inclusion/exclusion criteria. At this point, 371 articles were excluded, yielding a total of 28 studies to be included in this integrative review. All steps in the selection process were conducted by the first and last authors independently. The reason for retaining or excluding articles was discussed between them until consensus was reached. If there was disagreement about which articles should be included, these articles were read by the other two co-authors and then inclusion or exclusion was determined. The process of selection for the inclusion of articles is summarized in Fig. 1.

### Quality appraisal

The first and last authors independently assessed the quality of the 28 included articles with an instrument for appraising methodologically heterogeneous articles developed by Hawker et al. [27]. The quality appraisal explored nine components: (I) title and abstract, (II) introduction and aims, (III) method and data, (IV) sampling, (V) data analysis, (VI) ethics and bias, (VII) results, (VIII) transferability or generalizability, and finally (IX) implications and usefulness. Each article was assessed against predetermined criteria in the appraisal tool, where each criterion was rated on a scale from 1 (very poor) to 4 (very good). The scores for each assessment were added to obtain an overall total score and rating, ranging from very poor (9) to very good (36) [27]. A total score of less than 50% of the maximum (i.e., < 18) indicated that an article was considered to be of poor to very poor quality. The minimum score of the included articles in this integrated review was 19, and the maximum score was 35 (see Table 4). Hence, no articles were excluded based on the quality appraisal.

**Table 4:** Overview of the quality appraisal scoring of the articles included in the result

First author (year), country of origin [reference number]	Quality appraisal^a^									Total score^b^
	I	II	III	IV	V	VI	VII	VIII	IX	
Armstrong (2000), UK [40]	4	3	3	3	1	1	2	2	3	22
Batanero (2019), Spain [52]	3	3	3	2	3	2	4	2	3	25
Bloeming-Wolbrink (2018), Netherlands [28]	4	4	4	3	3	3	3	3	3	30
Borg (2001), Sweden [44]	3	4	3	1	3	1	3	1	1	20
Bourquin (2008), USA [43]	2	2	4	2	4	1	4	2	3	24
Bracken (2014), Ireland [32]	4	4	4	3	4	1	4	3	4	31
Cantin (2019), Canada [37]	4	4	4	3	4	3	4	3	3	32
Carrera (2017), Spain [34]	4	4	4	3	4	3	4	3	4	33
Côté (2013), Canada [55]	4	3	4	3	3	3	4	3	3	30
Damen (2014), Netherlands [29]	3	4	4	4	4	4	4	4	3	34
Damen (2015), Netherlands [30]	1	4	4	4	4	4	4	4	4	33
Demchinsky (2019), Russia [50]	3	2	4	3	3	1	4	2	3	25
Dufor (2005), Canada [48]	3	3	3	2	2	1	3	2	2	21
Evers (2012), Canada [39]	1	2	2	3	2	3	3	3	2	21
Franklin (2000), USA [42]	4	2	3	3	3	1	3	3	3	25
Garcia-Crespo (2018), Spain [53]	4	3	2	3	3	1	3	3	3	25
Gibson (2020), Norway [31]	2	2	3	3	2	1	3	2	3	21
Gibson (2018), UK [41]	3	4	4	3	1	1	4	3	3	26
Hansen (2004), USA [54]	3	4	3	3	2	1	3	2	4	25
Hartel (2017), Netherlands [38]	4	4	4	4	4	3	4	4	4	35
Hussain (2019), Pakistan [35]	4	3	3	3	3	1	3	2	3	25
Laby (2018), USA [49]	4	4	4	3	3	1	4	2	3	28
Lancioni (2010), Italy [45]	4	3	3	3	3	1	3	2	3	25
Nadal (2018), Spain [51]	4	3	3	3	2	1	3	2	3	24
Ogrinc (2018), UK [36]	4	4	4	3	4	3	4	3	4	33
Ranjbar (2013), Sweden [46]	4	4	4	3	3	3	4	3	4	32
Shivakumar (2014), India [33]	3	3	2	3	3	1	2	1	1	19
Vincent (2013), Canada [47]	4	4	3	4	3	4	4	4	4	34

^a^ Quality appraisal following the nine components as outlined by Hawker et al. [24]: I = title and abstract, II = introduction and aims, III = method and data, IV = sampling, V = data analysis, VI = ethics and bias, VII = results, VIII = transferability/generalizability, IX = implications and usefulness. Each article was assessed against predetermined criterions in the appraisal tool which were rated on a scale from 1 (very poor) to 4 (very good)

^b^ The scores for each assessment adds together to an overall total score and rating, ranging from very poor (9 = minimum score) to very good (36 = maximum score)

### Data extraction and synthesis

Data from the articles that were included and subsequently assessed for quality were entered into a summary table (see Table 5). The key outcomes from each article were compared with each other and thereafter deductively categorized and synthesised based on three of the main consequences of DB defined in the Nordic definition of deaf blindness. These are consequences related to *communication*, *orientation and the ability to move around freely and safely* and *access to information* [3]. A fourth category, *psychological adaptation to deafblindness*, was added to the categorization.

**Table 5:** Key outcomes of the included articles presented in relation to their categorization

Author(s) (year), country of origin [reference number]	Evaluated intervention	Aim of evaluated intervention	Key outcome	Persons with DB^i^ 18-65 years (n)	Diagnosis
Main focus: Communication					
Armstrong and Heidingsfield (2000), UK [40]	Aromatherapy and therapeutic massage	To promote confidence and communication as well as enhancing a sense of well-being with aromatherapy and therapeutic massage.	Aromatherapy and therapeutic massage increased tactile communication with others. The aromatherapy and therapeutic massage seemed to make the person with DB more sociable, outgoing, and more acceptant to tactile contact thus enhancing communication skills and confidence.	1	CDB^ii^
Bloeming-Wolbrink et al. (2018), Netherlands [28]	Intervention programme for interaction and bodily emotional traces (BET)	To examine the effects of a two-phase intervention program for caregivers working with adults with CDB and intellectual disability aimed at fostering harmonious interactions (Phase I), and the use and recognition of participant expressions based on a BET (Phase II).	Occurrence of all target behaviours across participants increased during the intervention; participant expressions based on BET increased after the interaction practicing and increased further after the BET practicing.	5	CDB
Bracken and Rohrer (2014), Ireland [32]	Picture Exchange Communication System (PECS)	To facilitate for adults with DB and learning disabilities to use an adapted form of PECS. Additional aims were to measure its efficacy using the percentages of independent requests and to assess if it is possible to generalise acquired skills to other contexts and communicative partners.	All participants demonstrated higher levels of independence in requesting after PECS practice. The acquired skills could be generalised to other contexts and communication partners.	3	CHARGE syndrome, Congenital Rubella syndrome
Cantin et al. (2019), Canada [37]	Communication Assistive Technology (CAD) based on a braille display note taker connected to a Smartphone via Bluetooth	To compare the communication in real-life situations and how these interactions work with or without CAD, and to describe the participant’s emotional experience after using the CAD.	CAD is useful and modifies the nature of interaction and communication. Although the use provided positive attitudes there were some recurring technical problems, which implies that CAD needs to be developed further.	1	Usher syndrome
Carrera et al. (2017), Spain [34]	Vibrotactile glove	To assess the viability of communication through a vibrotactile device.	After practicing, the overall message identification rate was 97%.	4	Usher syndrome
Damen et al. (2014), Netherlands [29]	Educational intervention (High-Quality Communication [HQC]) for social partners	To investigate the instrumental value of the theory of intersubjectivity for understanding and stimulating interpersonal communication.	HQC increased the rate of intersubjective behaviours. This was most evident in the dyadic interaction between the participant and the social partner.	1	Goldenhar syndrome
Damen et al. (2015), Netherlands [30]	HQC for social partners	To test the effects of the HQC intervention on forms of intersubjectivity in interpersonal communication.	Some intersubjective behaviours increased. The findings indicated that the support of both attunement strategies and meaning-making strategies could have positive effects on the intersubjective communication.	4	Congenital rubella syndrome, premature birth
Evers et al. (2012), Canada [39]	Accessibility to telephone services	To describe the process in which the participant’s and rehabilitation agency’s goal was to make telephone services accessible.	As the client’s vision and hearing declined over time, accessibility to telephone services had to be adapted regularly to meet the needs of the client. This process was facilitated by a multiprofessional cooperation within the rehabilitation team.	1	Charcot-Marie-Tooth disease
Gibson et al. (2020), Norway [31]	Learning through meaningful outdoor activities	To design an activity that was possible to understand through the tactile sense, creating the best possibilities for language development.	Language of the participant with DB developed. Success factors were authenticity and meaningfulness of the activities	1	CHARGE syndrome
Gibson and Nichols (2018), UK [41]	Theoretical and practical framework for integrating autobiographical memory with experiential learning outdoors	To describe the relationship between autobiographical memories and outdoor activities.	Outdoor education approach can aid in providing shared authentic memorable experiences and enhancing the construction of autobiographical memories in bodily tactile modality.	1	Congenital Rubella syndrome
Hartel et al. (2017), Netherlands [38]	Advanced signal processing on binaural hearing aids with nonlinear and linear amplification programs	To investigate if advanced signal processing of sound by hearing aids has any effect on speech intelligibility in noise and on sound localisation.	The linear program was used significantly more often than the nonlinear program (77% vs. 17%). There was no significant difference for speech intelligibility in noise and sound localisation between amplification types. The participants reported higher outcomes on ‘ease of communication’ and overall benefit, and significant lower on disability in predetermined and personally relevant situations when comparing the hearing aids worn in this study with their previously worn hearing aids with compression amplification.	18	Usher syndrome type 2a
Hussain et al. (2019), Pakistan [35]	Mobile application to assistive Muslim prayer (SmartPrayerAid)	To evaluate the assertiveness of the SmartPrayerAid when participating in Muslim prayer.	The result showed that SmartPrayerAid made it possible for the participants to concentrate on religious spirituality. It also facilitated participants in remaining synchronised with the Imam’s movements without the help of others.	3	Not defined
Ogrinc et al. (2018), UK [36]	Haptic interface to practice horseback riding	To evaluate a tactile wireless interface used when horseback riding in terms of the equestrian’s independence, confidence, enjoyment, comfort and safety.	The wireless interface enabled the equestrian to perceive the instructions given and to follow them. The equestrian experienced it joyfully and felt more secure and independent when using the device.	1	Not defined
Shivakumar et al. (2014), India [33]	A vibrotactile glove designed for communication (Braille glove vibration system)	To test if the Braille glove vibration system is useful in coding conversation in English text to Braille.	The results show that it was challenging for the participants to decode letters and digits. They were unable to decode words or sentences.	2	Not defined
Main focus: Orientation and the ability to move around freely and safely					
Borg et al. (2001), Sweden [44]	Eyeglasses with three microphones and four vibrators used for real-time directional analysis of sound sources	To test the eyeglass system.	Vibratory signals were easy, or relatively easy, to perceive and the participants could identify sound direction.	2	Not defined
Bourquin and Moon (2008), USA [43]	Communication cards for pedestrians to get assistance to cross a street	To compare if there was any difference between two communication cards, one small and one large, when asking for help to cross a street.	The larger communication cards were more efficient when soliciting help to cross a street.	7	Not defined
Demchinsky et al. (2019), Russia [50]	Rehabilitation programme (Second Sight Program) after Argus II retinal prosthesis surgery	To propose a new method for evaluating the functional results after implantation of an Argus II retinal prosthesis, and to describe the Second Sight Program and psychological assessment.	After the surgery and Second Sight Program, the person could discern colours and objects. The person also described a higher level of well-being, better health, feeling more independent and more self-confidence.	1	Usher syndrome
Dufour et al. (2005), Canada [48]	Auditory localisation training program (Auditory Localisation Evaluation System [SELA])	To evaluate if SELA might improve auditory skills useful for mobility and increase the feeling of security in travel situations.	The participant described more confidence in the capability of localisation.	1	Usher syndrome type 2
Franklin and Bourquin (2000), USA [42]	Assistance cards for street crossing	To compare the results obtained from the use of two different assistance cards to get assistance in crossing a street.	There was no significant difference between the two cards. Both were generally effective to receive assistance in crossing a street.	5	Not defined
Laby (2018), USA [49]	Sports vision training	To evaluate if sports vision training might improve objective and subjective visuomotor function.	After practicing, a 27 to 31% improvement in hand-eye coordination was achieved. There was also a 41% improvement in object tracking and visual concentration. After the intervention period, the person experienced a subjective improvement of her visual ability.	1	Usher syndrome type 3
Lancioni et al. (2010), Italy [45]	Orientation technology for indoor travelling in a wheelchair based on vibratory (direction) cues	To assess an adapted orientation technology developed for promoting correct direction and room identification during indoor travelling.	The participant was able to use the technology successfully when ambulating and could find the right direction and appropriate room entrances.	1	Traumatic brain injury
Nadal and Iglesias (2018), Spain [51]	Rehabilitation after retinal implant Argus II	To describe visual outcomes and posterior rehabilitation of a person receiving an Argus II prosthesis.	After the intervention, the person communicated more fluently with sign language. The vision had been improved and the participant could, among other things, read capital letters with high contrast and read sign language at a distance.	1	Usher syndrome type 2
Ranjbar and Stenström (2013), Sweden [46]	Vibrotactile aid for environmental perception (Monitor)	To test Monitor and compare the omnidirectional microphone with the directional microphone concerning the ability to detect, identify, and recognise the direction of sound-producing events.	Monitor improved the ability to detect, identify, and recognise the direction of sound producing events, although it was easier to detect than to identify the sounds. The omnidirectional microphone got better scores in home environment, whereas the directional microphone scored better in traffic.	4	Usher syndrome type 1
Vincent et al. (2013), Canada [47]	Electronic mobility aid devices (Breeze and MiniGuide)	To assess performance and satisfaction with independent mobility and the technical aid provided with Breeze and MiniGuide, and to assess the use of the electronic mobility aid devices.	The persons benefited from Breeze and MiniGuide, respectively, and they were satisfied with the use. Nevertheless, the persons felt that both Breeze and MiniGuide needed further development.	2	Usher syndrome
Main focus: Access to information					
Batanero et al. (2019), Spain [52]	An adapted digital educational platform: Moodle	To assess the learning performance when engineering students used a non-adapted learning platform and the adapted learning platform.	The students learning significantly improved when using the adapted learning platform.	3	Not defined
Garcia-Crespo et al. (2018), Spain [53]	A technical device (GoAll) developed to increase the autonomy of individuals with DB	To evaluate if GoAll allows people with DB to get direct access to content broadcasted on digital television.	Out of the participants 55% reported that they used GoAll somewhere between 5 to 7 Days a week watching news, movies, documentaries, reality shows, and entertainment shows. Eight of them (89%) found it easy to use GoAll independently. They experienced greater autonomy, which led to a sense of satisfaction. They became more motivated to discuss and exchange experiences with others about TV shows they had watched.	9	Not defined
Hansen et al. (2004), USA [54]	Solution for reading a screen via screen reader or braille display (HTML-form System)	To evaluate HTML-form System in computer-based tests.	The participants stated that the HTML-form System was challenging to use in computer-based tests, but the person who used the Braille display considered himself to benefit from the program.	2	Not defined
Main focus: Psychological adaptation to deafblindness					
Côte et al. (2013), Canada [55]	Life Transitions Through Personal Goals program – a group intervention in in five stages	To increase psychological well-being, self-determination and ability to set, plan, and pursue a goal using learned strategies.	Result of the intervention showed partial significant positive effect on meaning of life. No significant changes were shown on serenity, self-determination and ability to set, plan, and pursue a goal.	7	Usher syndrome type 2

^i^ *DB* Deafblindness

^ii^ *CDB* Congenital deafblindness

The categorization was first performed individually by three of the authors (the first, third and last authors) and thereafter compared and discussed within the whole research group together with representatives from The Swedish National Resource Center for Deafblindness until consensus regarding the categories was reached.

## Results

In the results section, first demographic data of the articles are reported, followed by the synthesised key outcome of each article.

### Demographic data

In the current review, the geographical origin of the included articles was categorized according to the first author’s affiliation. Most of the included articles were conducted in Europe (*n* = 17), but there were also articles from North America (*n* = 9) and Asia (*n* = 2). No articles from Africa, Oceania, or South America were found. All included articles were written in English.

The participants with DB in the included articles had different diagnoses. The most common diagnoses were different types of Usher syndrome (types 1, 2, 2a, and 3). Other diagnoses were Charcot-Marie-Tooth disease, CHARGE syndrome, congenital Rubella syndrome and Goldenhar syndrome. In several of the included articles, DB was caused by injuries or premature birth or the cause was not reported (see Table 5). In some articles [28–31], the intervention was aimed at the staff.

### Synthesis of the key outcomes

In the section below, the key outcomes of the identified articles are presented following the main focus of the respective articles: *communication*, *orientation and the ability to move around freely and safely*, *access to information*, and *psychological adaptation to deafblindness* (see Table 5). Ten out of 28 articles had overlapping outcomes and thereby relate to several categories. Table 6 gives an overview of which categories the articles are focusing on; a capital X in bold indicate the key focus, whereas a lower case x indicates some outcome in relation to the category (see Table 6).

**Table 6:** Overview of included articles and the relationship between their interventions and the four categories^a^

First author (year) [reference number]	Communication	Orientation and the ability to move around freely and safely	Access to information	Psychological adaptation to deafblindness
Armstrong (2000) [40]	**X**			
Batanero (2019) [52]			**X**	
Bloeming-Wolbrink (2018) [28]	**X**			
Borg (2001) [44]		**X**		
Bourquin (2008) [43]	x	**X**		
Bracken (2014) [32]	**X**			
Cantin (2019) [37]	**X**			
Carrera (2017) [34]	**X**			
Côté (2013) [55]				**X**
Damen (2014) [29]	**X**			
Damen (2015) [30]	**X**			
Demchinsky (2019) [50]	x	**X**	x	
Dufour (2005) [48]		**X**		
Evers (2012) [39]	**X**			
Franklin (2000) [42]	x	**X**		
Garcia-Crespo (2018) [53]	x		**X**	
Gibson (2020) [31]	**X**			
Gibson (2018) [41]	**X**	x		
Hansen (2004) [54]	x		**X**	
Hartel (2017) [38]	**X**	x		
Hussain (2019) [35]	**X**	x		
Laby (2018) [49]		**X**		
Lancioni (2010) [45]		**X**		
Nadal (2018) [51]	x	**X**	x	
Ogrinc (2018) [36]	**X**	x		
Ranjbar (2013) [46]		**X**		
Shivakumar (2014) [33]	**X**			
Vincent (2013) [47]		**X**		

^a^ An capital X in bold indicate the key focus of the intervention in the article, whereas an lower case x indicates additional intervention outcome

### Communication

In the current review, fourteen articles focus mainly on access to communication. The interventions had various focuses such as tactile aids for communication, advanced signal processing on binaural hearing aids, accessibility to telephone services, interventions for tactile interaction, and education for staff to use tactile interactions.

One aid for communication was the Picture Exchange Communication System (PECS) [32]. In the intervention, adapted PECS cards were tested by three participants who had CHARGE syndrome or congenital rubella syndrome and additional learning disabilities. For two of them, the PECS cards consisted of black swell images on a white background attached to a piece of cardboard with Velcro. The third person had residual vision, thus the PECS cards consisted of large, close up, coloured pictures, laminated and with Velcro. In the intervention, the participants first practiced to associate specific items with the related card. In the following period, they practiced to make independent requests by using the cards and, in a later phase, to make themselves independent of their communicative partner and to develop persistence. The last step focused on picture discrimination of nonpreferred and preferred items. The results showed an increased level of independent requests, which the study participants could also generalize to other contexts and in communication with others.

In two articles, vibrotactile gloves were tested. One glove converted written text from a computer (using hardware control procedures and a screen input program) to a glove with six positions that could vibrate, equivalent to the Braille cell [33]. Two participants tested the glove and experienced that it was challenging to decode the vibration of letters and digits and that they were unable to decode the vibrations of words and sentences. In the second article, the vibrotactile glove conveyed written pre-recorded messages from a smartphone to vibrations of the glove fingers using different frequencies transmitted via a wireless link. Four participants with Usher syndrome practiced to recognize 20 pre-recorded messages [34]. The result demonstrated an overall message identification rate of 97% after practicing.

SmartPrayerAid is a mobile application to assist in Muslim prayer developed by Hussain et al. [35]. When the Imam led prayer, the spoken words were converted into vibrations to the application in a smartphone or a smartwatch through voice recognition. Three participants tested the device. The smartphone was placed in the participants’ front pocket of a shirt or a pair of trousers, while the smartwatch was worn on the right wrist. The participants thought it was easier to concentrate on religious spirituality with help from the SmartPrayerAid. The device also facilitated the participants synchronizing their movement with the Imam without the help of others.

A tactile wireless device to be used for communication when practising horseback riding was tested in a study by Ogrinc et al. [36] and evaluated by a participant with DB and Asperger syndrome. The device enabled the equestrian to perceive and follow vibrations as messages from the instructor via a mobile phone. The equestrian had a remotely controlled wireless interface attached to his arms to receive the messages. The participant experienced it joyful to use the device, and felt more secure and independent when horseback riding.

One participant with Usher syndrome type 1 tested the Communication Assistive Technology (CAD) developed by Cantin et al. [37]. CAD was based on a Braille display note taker connected to a smartphone via Bluetooth. With the technology, the participant read in Braille what was communicated through the smartphone. In the study, communication in real-life situations with CAD was compared with situations without it. The results of the study showed that the CAD device was useful and could modify the nature of interaction and communication, although with some recurring technical problems.

A study by Hartel et al. [38] included 18 participants with Usher syndrome type 2a. The participants evaluated advanced signal processing binaural hearing aids, which had two different amplification programs: a nonlinear and a linear program. The results showed that the linear program was used significantly more often than the nonlinear program (77% vs. 17%). There was no significant difference for speech intelligibility in noise and sound localization between the amplification programs. When comparing the hearing aids tested in the study with previously worn hearing aids, the participants reported higher outcomes in overall benefit with the tested hearing aids and ‘ease of communication’. They also scored significantly lower on disability in predetermined and personally relevant situations.

Evers et al. [39] described a rehabilitation process to make telephone services accessible. The participant had Charcot-Marie-Thoot disease, and due to the progressive decline of vision and hearing, was in need of recurrent adaptations to be able to use telephone services. Initially he could manage calls received by reading text, but later on the text had to be converted to Braille. Different devices and systems were used during the process, which was facilitated by cooperation of the multiprofessional rehabilitation team.

Two articles described tactile interactive interventions. One study of aromatherapy and therapeutic massage [40] and one of learning through meaningful outdoor activities [41].

The article by Armstrong and Heidingsfeld [40] described a case study with a participant with CDB. The aim of the study was to evaluate how aromatherapy and therapeutic massage could enhance self-confidence, communication and well-being. After the intervention, the participant seemed more sociable, outgoing, and more comfortable with tactile contact. Her communication skills and confidence had increased.

One article focused on learning in outdoor activities for a person who had congenital Rubella syndrome. In the study, Gibson and Nicholas [41] identified that outdoor education can enhance shared authentic memorable experiences and improve the construction of autobiographical memories in bodily tactile modality. Both communication and orientation outdoors were facilitated.

Four publications described intervention programmes for staff (i.e., social partner’s, teachers or caregivers) to improve communication with people with DB. An educational intervention; High-Quality Communication (HQC) was described in two publications [29, 30], with one article focused on teaching communication and pedagogical skills for support staff and teachers [31] and one article describing an “intervention programme for interaction and bodily emotional traces” (BET) [28]. One person with Goldenhar syndrome attended a pilot study aimed at understanding and stimulating interpersonal communication [29]. Social partners participated in education regarding interaction with the person with DB. The results of the study showed increased rates of intersubjective behaviours, e.g., dyadic interaction and shared emotions, between the person with DB and the social partners [29]. In a later study of HQC, the effect of the intervention was tested [30]. Four people with DB resulting from congenital rubella syndrome or premature birth participated in the study. The results showed that the four persons’ intersubjective behaviours increased. The results also indicated that the support of attunement strategies and meaning-making strategies could have positive effects on intersubjective communication.

In a study by Gibson et al. [31] an intervention was developed to facilitate communication. One participant with CHARGE syndrome was involved in the intervention. The intervention consisted of teaching supportive staff and teachers communication and pedagogical skills, using video recordings and analysis, as well as collaboration between staff. The results of the study showed that the language of the participant with DB further developed. Four elements to achieving language development were identified: authenticity and meaningfulness of activities, use of video recordings and analysis of the video footage, collaboration between participants with different skills and backgrounds throughout the project, and close links between practice and theory.

Bloeming-Wolbrink et al. [28], have conducted a study that involved five people with CDB and intellectual disability. The study was composed of BET in a two-phase intervention program for caregivers. The program consisted of coaching, video recordings and feedback to caregivers. The goal was to foster harmonious interactions and to recognize the expressions of people with DB. The overall result of the study showed that both the target behaviours and the recognized expressions from the people with CDB increased, although changes were minimal.

### Orientation and the ability to move around freely and safely

Several types of interventions described in the articles had their focus on *orientation and the ability to move around freely and safely*. Two of the articles describe assistance cards for street crossing, four articles technical aids, and four articles rehabilitation programs after either cochlear implant (CI) or retinal prosthesis surgery.

Franklin and Bourquin [42] described the use of communication cards for getting help to cross a street. In this study, five participants tested and evaluated two different cards: one card with text and one card with the same text but with an icon added illustrating one person supporting another. The result showed no significant difference between the cards to get assistance to cross a street – both were generally effective. Later, Bourquin and Moon [43] compared whether there were differences in obtaining assistance to cross the street by using a small card compared with a larger one with the same information. In this study, seven participants were enrolled, and the results showed that the larger card was more efficient when soliciting help to cross a street.

Four articles that investigated the use of technical aids for orientation and the ability to move around freely and safely relied on vibrotactility. In an experimental study by Borg and Rönnberg [44], two participants tested eyeglasses equipped with microphones and vibrators used for real-time directional analysis of sound sources. The results of the experiment showed that the vibration signals were easy, or relatively easy, for one of the participants to perceive. It was possible for both participants to identify the sound direction.

Lancioni et al. [45] tested orientation technology for promoting correct direction and room identification during indoor travelling for an adult with DB using a wheelchair. The orientation system consisted of a portable control unit with vibrating boxes presenting vibratory (direction) cues. The device was stored inside a pocket at the back of the wheelchair. The vibrating boxes were connected to optic sensors in a hallway. The results showed that the participant was able to ambulate and find the right direction and the appointed rooms in a hallway.

Four participants with Usher syndrome type 1 tested the vibrotactile aid Monitor for environmental perception [46]. Monitor consists of a mobile phone containing an application, an external microphone, an amplifier, and a vibrator. The microphone and the vibrator were connected to the mobile phone via a headset. In the study, an omnidirectional microphone was compared with a directional microphone to detect, identify and recognize the direction of sound. The results showed that Monitor improved the participants’ ability to detect, identify and recognize the direction of sound-producing events. The results also showed that the participants found it easier to detect the sounds than to identify them. The participants experienced that the omnidirectional microphone was more functional at home but that the directional microphone functioned better in environments with traffic.

Two electronic orientation aid devices, Breeze and MiniGuide, were tested by two people with Usher syndrome [47]. One participant tested Breeze and one MiniGuide. Breeze is a global positioning system (GPS) with a speech function that auditorily speaks the name of streets or places, whereas MiniGuide is a vibration device based on eco-localization for detecting obstacles in the environment. The results showed that both participants were able to move around freely in the environment with assistance from the devices. The participants were satisfied with the use of the devices, although they pointed out that the devices needed to be further developed.

Rehabilitation programmes for auditory localization and improving vision or hearing after CI or retinal surgeries were evaluated in four articles.

The Auditory Localization Evaluation System (SELA), a rehabilitation programme developed to improve auditory skills via a computer after a CI, was evaluated by one participant [48]. In the programme, the participant was encouraged to localize pre-recorded sounds from three positions. The participant, sitting on a swivel chair, practices localizing the sound by facing the source. In this study, a woman with Usher syndrome type 2 who had received a CI attended SELA to improve her auditory skills for orientation and increase her feeling of security when travelling. After attending SELA in two periods of 21 sessions in total, she felt more confident in the capability of sound localization.

To improve vision, one person with Usher syndrome type 3 who had received a CI evaluated a sports vision training programme for 14 weeks to increase objective and subjective visuomotor function [49]. After practicing object tracking and visual concentration, a 27 to 31% improvement in hand-eye coordination was achieved. There was also a 41% improvement in object tracking and visual concentration. Furthermore, the person experienced a subjective improvement of visual ability.

Two articles [50, 51] described rehabilitation programs after Argus II retinal prosthesis surgery. Two participants with Usher syndrome underwent similar rehabilitation programs (in one study the type of Usher was not stated [50], whereas in the other the participant has Usher type 2 [51]). In the study by Demchinsky et al. [50] the participant evaluated the functional results after implantation and the rehabilitation programme Second Sight and psychological assessment. After attending the Second Sight programme the participant could discern colours and objects, and described a higher level of well-being, better health, feeling more independent and more self-confident. In the second study, Nadal and Iglesias [51] described visual outcomes after rehabilitation. The results showed that the participant communicated more fluently with sign language at a distance. The participant had also improved vision and could, among other things, read capital letters with high contrast after the intervention.

### Access to information

In the current review, three articles primarily relate to * access to information*. The articles describe devices or programs that convert either written text, sound and/or speech to Braille or text that was read out loud via a computer or another device.

In a study by Batanero et al. [52], three engineering students with DB tested and compared a nonadapted digital educational platform with an adapted platform (Moodle). The platforms were tested in three learning tasks. The tasks were performed via a platform that complied with the standards “Access for All” recommendations and compared with an adapted Moodle platform that enabled extensive descriptions of images and audio, screen reader and device, which translates text to Braille. The adapted Moodle platform was installed with regard to individual needs and preferences. The results showed that the students’ learning was significantly improved when using the adapted learning platform.

A technical device, GoAll, was designed to offer people with DB direct access to content broadcasted on digital television. GoAll was evaluated by nine participants with DB in a study by García-Crespo et al. [53]. The device allowed reading of text from TV, closed captions, with either a mobile device or a Braille display. Overall, the participants were satisfied with GoAll and used it frequently. They reported greater autonomy and became more motivated to discuss and exchange experiences with others about TV shows they had watched.

The third study concerning access to information tested a solution for reading from a screen via a screen reader or Braille display – a so-called HTML-form System [54]. Two participants evaluated the system: one by using a screen reader and one by reading Braille. The participants found the system challenging to use due to difficulties with the various navigation modes of the screen reader. However, the person who used the Braille display reported that he felt he benefited from the system.

### Psychological adaptation to deafblindness

The article written by Côte et al. [55] describe a group intervention based on Life Transitions Through Personal Goals program focusing on *psychological adaption to DB*. Seven participants with Usher syndrome Type 2 attended the five-step program, which consisted of 13 meetings. The goals of the programme were to increase psychological well-being, self-determination and the ability to set, plan, and pursue a goal. The goals were measured with questionnaires pre- and post-intervention evaluating such variables as the meaning of life, the feeling of serenity and self-determination, and the ability to set, plan, and pursue a goal. The results of the intervention showed a partially significant positive effect on the variable meaning of life. No significant changes were shown in the other variables.

## Discussion

This integrated review offers insight into the research of evaluated interventions for people with DB conducted during the last two decades. A vast majority of the interventions identified in the articles relate to communication, followed by interventions to facilitate orientation and the ability to move around freely and safely. Three articles focus primarily on access to information, and only one on psychological adaptation to DB. However, no article specifically focusing on intervention for limitations in social life or social support was found. This result is surprising, as it is generally reported that DB may lead to isolation [2, 8, 56, 57] and stress [13, 58] and may have a negative impact on mental health [8–10, 58, 59]. Nevertheless, although these aspects were not primary outcome measures of any of the included studies, it is likely that interventions aiming at communication, access to information, orientation and the ability to more around freely and safely, and psychological adaptation to DB could have a positive impact on limitations in social life and thus also on health and isolation.

In this integrated review, many of the identified interventions targeted people with DB, but there were also interventions that focused on teaching the staff how to best accommodate DB. Both of these perspectives are, according to the Nordic definition of deafblindness [3], important to consider to meet the needs of people with DB with regard to activity and full participation in society [15].

Two aspects implicitly identified in a few of the included articles were the need for adequate instruction and practise to master use of or adjustment to the new devices or interventions [60, 61]. It is important to provide proper instruction and adequate time for practise of interventions to enable success in their use for people with DB, both in research and in rehabilitation praxis. The rapid development within the area of information and communication technology can be a limiting factor in that people with DB continually need access to and practicing in the latest technology [58, 62, 63]. In light of the COVID-19 pandemic and expanded digitalisation during recent years, it would be of interest to create and evaluate interventions aimed at accessible information for people with DB using digital media, both for everyday use and during extraordinary circumstances, such as during pandemics and disasters [64].

The review identified some gaps in research focus: for example, only two identified studies [39, 55] focused on the decline in function over time. One of the studies was a descriptive case study [39], whereas the other one was a group intervention with a focus on redirection of life and the possibility of setting new goals [55]. There were three articles presenting case reports from a naturalistic setting, involving only one person per report, and no article at all evaluating existing regular rehabilitation services. It has been argued that there is a need to evaluate methods used in rehabilitation settings to be cost-effective and of high quality. This has also been addressed in relation to the need for research on interventions for people with DB [65]. In line with Wittich and colleagues [66], we think that rehabilitation practise could be improved by an increased number of evidence-based interventions.

The included research typically involves a small number of participants, conducted within one country. As DB is a rare condition [1, 5], it could be difficult to recruit participants in one region or country. It is common that people with DB can experience fatigue [18, 67], and there are risks that repeated study participation can add to this burden. Despite the risk with involving people with DB in research, the target group they themselves have to evaluate interventions aiming at them, which is why their participation in research is essential. It is further important that people with DB are involved in the process of developing interventions that address their needs, which has been previously addressed in several studies [65, 68–72]. Consideration of how to involve people with DB in research procedure needs to be taken into account. The process have to be adapted and considered with respect to each personal needs for those who participate in research [65]. It is possible that an international collaboration could limit the burden of participation in research, and this would allow for a varied sample of people living with DB. Enhanced international research collaboration could also contribute to larger study samples and would most certainly improve the quality, as well as increase the generalization of future findings.

Overall, the small number of studies is problematic, and calls for more research studies that focus on interventions in order to build evidence-based rehabilitation. Until then, our current compilation of research can offer some guidance and conclusions, as well as stimulate future replications. Further intervention studies could, for example, include structured ways to deal with changed communication modalities, psychological processes and new strategies for orientation and the ability to move around freely and safely. We did not identify any articles focusing on support for family members, although DB affects the whole family [17, 19, 73, 74], which is an area that needs to be investigated in the future.

### Method discussion

This integrated review summarizes the findings regarding interventions for adults with DB that have been evaluated by themselves, their relatives or professionals, published in peer-reviewed journals from 2000 to 2020. All steps in the review were systematically performed, and by providing the search strategies used in each database, the reliability of the results was strengthened. A thorough description of the systematic search process as well as the adoptation of a well-established design by Whittemore [23] have strengthened the quality of this review. This, together with that the screening, quality appraisal and data extraction was performed independently by two researchers, ensures consistency in the conformity of inclusion criteria and reduce the risk for systematic errors [75]. Literature reviews have limitations regarding inconsistent search terminology, which might result in covering only 50% of eligible articles, and it has been recommended that at least two to three search strategies be used to identify the maximum number of articles [23]. Thus, two experienced librarians carried out a comprehensive database search, which was combined with a manual search for additional articles not available in the systematic search, thereby increasing the validity of the results. A possible limitation of the review is that one inclusion criterion was that articles be written in Danish, English, Norwegian or Swedish, which have led to the exclusion of articles reporting interventions for people with DB if written in any other language. Articles that only focused on medical interventions without any accompanying rehabilitation were also excluded, which led to the exclusion of 20 articles describing surgical interventions or audiological evaluations with regard to CI. The exclusion of these articles can be seen as a call for researchers to not only describe medical interventions but also the subsequent rehabilitation processes in future research. We know that there are numerous interventions for people with DB used in clinical praxis. Such interventions might not be documented in the scientific literature, but are more often presented in experience based literature and shared at conferences aimed at clinicians. Thus we did not find them in our systematic searches, using specific search terms.

## Conclusion

There are a limited number of scientifically documented interventions for people with DB that have been evaluated by either themselves, their relatives or professionals. Technical devices to facilitate communication were the most common interventions identified. Most of the existing articles involved one to five participants with DB, and only four studies involved more participants. More studies with a larger number of participants are needed, which could be facilitated by international cooperation between practitioners, researchers and people with DB. We therefore invite clinicians and researchers to join hands in documenting, evaluating and publishing interventions from praxis in research journals in order to build and advance the evidence based and promising practices to support people with DB worldwide.

## Supplementary Information

**Additional file 1.**

## Data Availability

The data that support the findings of this study are available from the corresponding author but restrictions apply to the availability of these data, which were used under license for the current study, and so are not publicly available. Data are however available from the authors upon reasonable request and with permission of the corresponding author. Overview of the search terms and search strategies used in each database is found in additional file 1.

## Abbreviations

BET: bodily emotional traces
CAD: communication assistive technology
CDB: congenital deafblindness
CI: cochlear implant
DB: deafblindness
GPS: global positioning system
HQC: high-quality communication
PECS: picture exchange communication system
SELA: auditory localization evaluation system
