# Supplementary material for: Interventions for adults with deafblindness - an integrative review
Source: BMC Health Serv Res. 2022 Dec 30;22:1594. doi: 10.1186/s12913-022-08958-4 (PMC9801155; doi:10.1186/s12913-022-08958-4)
Supplement: Supplementary file 1 — Additional file 1. [file 12913_2022_8958_MOESM1_ESM.docx]

Additional file 1

| Overview of the search terms and search strategies used in each database | |
| --- | --- |
| **AMED** | |
| **Search 1 (S1)**  **Deafblindness** | deafblindness OR deaf-blindness OR deafblind OR deaf-blind OR "dual sensory" |
| **Search 2 (S2)**  **Syndromes** | - |
| **Search 3 (S3)**  **Vision** | "vision impairment" OR "vision impaired" OR "vision loss" OR "visual loss" OR "visual impairment" OR "visual impaired" OR "visually impaired" OR retinitis pigmentosa OR blind OR blindness |
| **Search 4 (S4)**  **Hearing** | "hearing loss" OR "hearing impairment" OR "hearing impaired" OR "hard of hearing" OR "auditory impaired" OR "auditory impairment" OR deaf OR deafness |
| **Search 5 (S5)** | A combination of the searches S3 AND S4 |
| **Search 6 (S6)** | A combination of the searches S1 OR S2 OR S5 |
| **Search 7 (S7)**  **Intervention** | - |
| **Search 8 (S8)** | A combination of the searches S6 AND S7 |
|  | |
| **Cinahl** | |
| **Search 1 (S1)**  **Deafblindness** | [MH] "Deaf-Blind Disorders+"  OR  deafblind OR deaf-blind OR deafblindness OR deaf-blindness OR “dual sensory loss" OR "dual sensory impairment" OR "dual sensory impaired" OR "dual sensory impairments" |
| **Search 2 (S2)**  **Syndromes** | Usher syndrome, Charge syndrome, Alstrom syndrome, Wolfram syndrome, Refsum disease, Capos syndrome, Pharc syndrome |
| **Search 3 (S3)**  **Vision** | [MH] "Blindness+" OR [MH] "Vision, Subnormal"  OR  "vision loss" OR "vision impairment" OR "vision impaired" OR "visual loss" OR “visual impairment” OR "visual impaired" OR "visually impaired" OR retinitis pigmentosa |
| **Search 4 (S4)**  **Hearing** | [MH] "Hearing Disorders+"  OR  ”hearing loss” OR “hearing impairment” OR “hearing impaired” OR "hard of hearing" OR “auditory impairment” OR “auditory impaired” OR deaf OR deafness |
| **Search 5 (S5)** | A combination of the searches S3 AND S4 |
| **Search 6 (S6)** | A combination of the searches S1 OR S2 OR S5 |
| **Search 7 (S7)**  **Intervention** | [MH] "Rehabilitation+" OR [MH] "Education, Special+" OR [MH] "Communication Aids for Disabled+" OR [MH] "Sensory Aids+" OR [MH] "Interpreter Services"  OR  "rehabilitation" OR habilitation OR counseling OR "next of kin" OR spouse* OR partner* OR "special education" OR "communication aids" OR "hearing aid*" OR "cochlear implant*" OR "cochlea implant*" OR braille OR "sensory aid*" OR "sign language" OR assistive OR assistant* OR haptic* OR interpreter* OR vibrator* OR vibration* OR computer* OR technical OR mobility OR mobilize OR mobilise OR mobilization OR mobilization OR work OR employment |
| **Search 8 (S8)** | A combination of the searches S6 AND S7 |
|  | |
| **Embase** | |
| **Search 1 (S1)**  **Deafblindness** | 'deafblindness'/exp  OR  'deaf blind*' OR 'deaf?blind disorder*' OR 'deaf?blind*' OR 'dual sensory' |
| **Search 2 (S2)**  **Syndromes** | Usher syndrome, Charge syndrome, Alstrom syndrome, Wolfram syndrome, Refsum disease, Capos syndrome, Pharc syndrome |
| **Search 3 (S3)**  **Vision** | 'visual impairment'/exp OR 'low vision'/exp OR 'visually impaired person'/exp OR 'retinitis pigmentosa'/exp  OR  [ti,ab,kw] blind OR 'low vision' OR 'vision loss' OR 'visual loss' OR 'visual impairment' OR 'vision impairment' OR 'visually impaired' 'retinitis pigmentosa' |
| **Search 4 (S4)**  **Hearing** | 'hearing impairment'/exp OR 'hearing impaired person'/exp  OR  [ti,ab,kw] 'hearing loss' OR deaf* OR 'hearing impair*' OR 'auditory impair*' OR 'impaired hearing' OR 'hard of hearing' |
| **Search 5 (S5)** | A combination of the searches S3 AND S4 |
| **Search 6 (S6)** | A combination of the searches S1 OR S2 OR S5 |
| **Search 7 (S7)**  **Intervention** | 'rehabilitation'/exp OR 'family'/exp OR 'social support'/exp OR 'special education'/exp OR 'nonverbal communication'/exp OR 'audiovisual aid'/exp OR 'communication aid'/exp OR 'sensory aid'/exp OR 'work'/exp OR 'vocational education'/exp OR 'occupation'/exp OR 'employment'/exp  OR  [ti,ab,kw] rehabilita* OR habilita* OR counsel* OR 'next of kin' OR spouse* OR partner* OR educat* OR 'special education' OR 'sign language' OR communicat* OR braille OR 'hearing aid* OR 'cochlear implant*' OR 'cochlea implant*' OR 'social service*' OR support OR assist OR 'occupational therapy' OR 'physical therapy' OR 'physiotherapy' OR work OR occupation OR employment OR vocation* OR interpreter* OR mobility OR 'audiovisual aids' OR 'visual aids' OR 'technical aids' OR ophthalmologist |
| **Search 8 (S8)** | A combination of the searches S6 AND S7 |
|  | |
| **ERIC** | |
| **Search 1 (S1)**  **Deafblindness** | deafblind OR deaf-blind OR deafblindness OR deaf-blindness OR "dual sensory loss" OR "dual sensory impairment" OR "dual sensory impaired" OR "dual sensory impairments” |
| **Search 2 (S2)**  **Syndromes** | Usher syndrome, Charge syndrome, Alstrom syndrome, Wolfram syndrome, Refsum disease, Capos syndrome, Pharc syndrome |
| **Search 3 (S3)**  **Vision** | "vision loss" OR "vision impairment" OR "vision impaired" OR "visual loss" OR “visual impairment” OR "visual impaired" OR "visually impaired" OR retinitis pigmentosa |
| **Search 4 (S4)**  **Hearing** | ”hearing loss” OR “hearing impairment” OR “hearing impaired” OR "hard of hearing" OR “auditory impairment” OR “auditory impaired” OR deaf OR deafness |
| **Search 5 (S5)** | A combination of the searches S3 AND S4 |
| **Search 6 (S6)** | A combination of the searches S1 OR S2 OR S5 |
| **Search 7 (S7)**  **Intervention** | Rehabilitation OR habilitation OR counseling OR "next of kin" OR spouse* OR partner* OR "special education" OR "communication aids" OR "hearing aid*" OR "cochlear implant*" OR "cochlea implant*" OR braille OR "sensory aid*" OR "sign language" OR assistive OR assistant* OR haptic* OR interpreter* OR vibrator* OR vibration* OR computer* OR technical OR mobility OR mobilize OR mobilise OR mobilization OR mobilisation OR work OR employment |
| **Search 8 (S8)** | A combination of the searches S6 AND S7 |
|  | |
| **PsychINFO** | |
| **Search 1 (S1)**  **Deafblindness** | [DE] "Deaf-Blind"  OR  [TI, AB] deafblind OR deaf-blind OR deafblindness OR deaf-blindness OR "dual sensory loss" OR "dual sensory impairment" OR "dual sensory impaired" OR "dual sensory impairments" |
| **Search 2 (S2)**  **Syndromes** | Usher syndrome, Charge syndrome, Alstrom syndrome, Wolfram syndrome, Refsum disease, Capos syndrome, Pharc syndrome |
| **Search 3 (S3)**  **Vision** | [DE] "Blind"  OR  [TI, AB] "vision loss" OR "vision impairment" OR "vision impaired" OR "visual loss" OR “visual impairment” OR "visual impaired" OR "visually impaired" OR “retinitis pigmentosa” |
| **Search 4 (S4)**  **Hearing** | [DE] "Hearing Disorders" OR [DE] "Deaf"  OR  [TI, AB ]”hearing loss” OR “hearing impairment” OR “hearing impaired”OR "hard of hearing" OR “auditory impairment” OR “auditory impaired” OR deaf OR deafness |
| **Search 5 (S5)** | A combination of the searches S3 AND S4 |
| **Search 6 (S6)** | A combination of the searches S1 OR S2 OR S5 |
| **Search 7 (S7)**  **Intervention** | [DE] "Rehabilitation" OR [DE] "Occupational Therapy" OR [DE] "Physical Therapy" OR [DE] "Psychosocial Rehabilitation" OR [DE] "Habilitation" OR [DE] "Hearing Aids" OR [DE] "Cochlear Implants"  OR  [TI,AB] Habilitation OR Rehabilitation OR Education OR “Communication aids” OR “sensory aid*” OR counceling OR “next of kin” OR spouse* OR partner* OR “hearing aid*” OR “cochlear implant*” OR “cochlea implant*” OR braille OR “sign language” OR assistive OR assistant* OR haptic OR interpreter* OR vibrator* OR vibration OR computer* OR technical OR mobility OR mobilize OR mobilise OR mobilization OR mobilization |
| **Search 8 (S8)** | A combination of the searches S6 AND S7 |
|  | |
| **PubMed** | |
| **Search 1 (S1)**  **Deafblindness** | Deafblind OR deafblindness OR deaf-blind OR deaf-blindness OR "dual sensory” |
| **Search 2 (S2)**  **Syndromes** | Usher syndrome, Charge syndrome, Alstrom syndrome, Wolfram syndrome, Refsum disease, Capos syndrome, Pharc syndrome |
| **Search 3 (S3)**  **Vision** | Vision, Low" [Mesh] OR "blindness" [Mesh] OR "Visually Impaired Persons" [Mesh] OR Retinitis pigmentosa [MeSH Terms]  OR  [Title/Abstract] "low vision” OR blindness OR "vision loss" OR "visual loss" OR "visual impairment" OR "vision impairment" OR “visually impaired” OR “retinitis pigmentosa” |
| **Search 4 (S4)**  **Hearing** | hearing impaired persons[Mesh] OR hearing loss [MeSH Terms]  OR  [Title/Abstract] “hearing loss” OR deaf OR deafness OR “hearing impairment” OR” hearing impaired” OR “auditory impaired” OR “auditory impairment” OR “impaired hearing” OR "hard of hearing" |
| **Search 5 (S5)** | A combination of the searches S3 AND S4 |
| **Search 6 (S6)** | A combination of the searches S1 OR S2 OR S5 |
| **Search 7 (S7)**  **Intervention** | Rehabilitation [MeSH Terms] OR Rehabilitation [MeSH Subheading] OR Family [MeSH Terms] OR Social support [MeSH Terms] OR Education, Special [MeSH Major Topic] OR Nonverbal Communication [MeSH Terms] OR Audiovisual Aids [MeSH Terms] OR Communication Aids for Disabled [MeSH Terms] OR Sesory Aids [MeSH Terms] OR Work [MeSH Terms] OR vocational education [MeSH Terms] OR occupations [MeSH Terms] OR employment [MeSH Terms]  OR  [Title/Abstract] rehabilitation OR habilitation OR counseling OR "next of kin" OR spouse OR spouses OR partner OR partners OR education OR "sign language" OR communication OR braille OR "hearing aids" OR "hearing aid" OR “cochlear implant" OR "cochlear implants" OR "cochlear implantation" " OR "cochlear implantations" OR "cochlea implant" OR "cochlea implants" OR "cochlea implantation OR "cochlea implantations" OR "social service" OR "social services" OR "support" OR "supportive" OR "assistance" OR assistant OR assistants OR assisted OR assistive OR "occupational therapy" OR "physical therapy" OR "physiotherapy" OR work OR occupation OR occupations OR employment OR vocational OR vocation OR vocations OR interpreter* OR mobility OR "audiovisual aids” OR "visual aids” OR "technical aids” OR ophthalmologist* |
| **Search 8 (S8)** | A combination of the searches S6 AND S7 |
|  | |
| **SCOPUS** | |
| **Search 1 (S1)**  **Deafblindness** | [TITLE-ABS-KEY] deafblind OR deaf-blind OR deafblindness OR deaf-blindness OR "dual sensory loss" OR "dual sensory impairment" OR "dual sensory impairments" OR "dual sensory impaired" |
| **Search 2 (S2)**  **Syndromes** | Usher syndrome, Charge syndrome, Alstrom syndrome, Wolfram syndrome, Refsum disease, Capos syndrome, Pharc syndrome |
| **Search 3 (S3)**  **Vision** | [TITLE-ABS-KEY] "vision loss" OR "vision impairment" OR "vision impaired" OR "visual loss" OR "visual impairment" OR "visual impaired" OR "visually impaired" OR “retinitis pigmentosa” |
| **Search 4 (S4)**  **Hearing** | [TITLE-ABS-KEY] "hearing loss" OR "hearing impairment" OR "hearing impaired" OR "hard of hearing" OR "auditory impaired" OR "auditory impairment" OR deaf OR deafness |
| **Search 5 (S5)** | A combination of the searches S3 AND S4 |
| **Search 6 (S6)** | A combination of the searches S1 OR S2 OR S5 |
| **Search 7 (S7)**  **Intervention** | [TITLE-ABS-KEY] "cochlea implant*" OR "cochlear implant*" OR "sensory aid*" OR "hearing aid*" OR "communication aid" OR "occupational therapy" OR "physical therapy" OR "physiotherapy" OR "activities of daily living" OR rehabilitation OR habilitation OR spouse* OR partner* OR "next of kin" OR work OR employment OR "social service" OR braille OR haptic* OR communication |
| **Search 8 (S8)** | A combination of the searches S6 AND S7 |
|  | |
| **Web of Science** | |
| **Search 1 (S1)**  **Deafblindness** | deafblind OR deaf-blind OR deafblindness OR deaf-blindness OR "dual sensory loss" OR “dual sensory impairment” OR "dual sensory impairments" OR "dual sensory impaired" |
| **Search 2 (S2)**  **Syndromes** | Usher syndrome, Charge syndrome, Alstrom syndrome, Wolfram syndrome, Refsum disease, Capos syndrome, Pharc syndrome |
| **Search 3 (S3)**  **Vision** | “retinitis pigmentosa" OR “visual loss" OR “visual impairment" OR "visual impaired" OR “visually impaired" OR "vision loss" OR "vision impairment" OR "vision impaired" |
| **Search 4 (S4)**  **Hearing** | ”hearing loss” OR “hearing impairment” OR “hearing impaired” OR "hard of hearing" OR “auditory impairment” OR “auditory impaired” OR deaf OR deafness |
| **Search 5 (S5)** | A combination of the searches S3 AND S4 |
| **Search 6 (S6)** | A combination of the searches S1 OR S2 OR S5 |
| **Search 7 (S7)**  **Intervention** | rehabilitation OR habilitation OR "occupational therapy" OR physiotherapy OR “physical therapy" OR "cochlea implant*” OR "cochlear implant*" OR "sensory aid*" OR "hearing aid*" OR "sign language" OR "communication aid*" OR interpreter* OR haptic* OR braille OR "sensory aid*" OR intervention* OR vibrator* OR vibration* OR computer* OR technical OR assistive OR assistant OR mobility OR mobilize OR mobilise OR mobilization OR mobilization OR education OR spouse* OR partner* OR "next of kin" OR work* OR employment* OR social OR functional OR training OR participation OR "activities of daily living" OR ADL OR communication OR support OR supportive |
| **Search 8 (S8)** | A combination of the searches S6 AND S7 |
